# Corneal dendritic cells in diabetes mellitus: A narrative review

**DOI:** 10.3389/fendo.2023.1078660

**Published:** 2023-01-27

**Authors:** Fengyi Liu, Chang Liu, Isabelle Xin Yu Lee, Molly Tzu Yu Lin, Yu-Chi Liu

**Affiliations:** ^1^ University of Cambridge, Girton College, Cambridgeshire, United Kingdom; ^2^ Singapore Eye Research Institute, Singapore National Eye Centre, Singapore, Singapore; ^3^ Tissue Engineering and Cell Therapy Group, Singapore Eye Research Institute, Singapore, Singapore; ^4^ Cornea and Refractive Surgery Group, Singapore Eye Research Institute, Singapore, Singapore; ^5^ Ophthalmology and Visual Sciences Academic Clinical Program, Duke-NUS Medical School, Singapore, Singapore; ^6^ Department of Ophthalmology, National Taiwan University, Taipei, Taiwan

**Keywords:** corneal dendritic cell, diabetic mellitus, corneal nerves, corneal epithelial cells, *in vivo* confocal microscopy, diabetic corneal neuropathy, diabetic microvascular complications, ocular surface

## Abstract

Diabetes mellitus is a global public health problem with both macrovascular and microvascular complications, such as diabetic corneal neuropathy (DCN). Using *in-vivo* confocal microscopy, corneal nerve changes in DCN patients can be examined. Additionally, changes in the morphology and quantity of corneal dendritic cells (DCs) in diabetic corneas have also been observed. DCs are bone marrow-derived antigen-presenting cells that serve both immunological and non-immunological roles in human corneas. However, the role and pathogenesis of corneal DC in diabetic corneas have not been well understood. In this article, we provide a comprehensive review of both animal and clinical studies that report changes in DCs, including the DC density, maturation stages, as well as relationships between the corneal DCs, corneal nerves, and corneal epithelium, in diabetic corneas. We have also discussed the associations between the changes in corneal DCs and various clinical or imaging parameters, including age, corneal nerve status, and blood metabolic parameters. Such information would provide valuable insight into the development of diagnostic, preventive, and therapeutic strategies for DM-associated ocular surface complications.

## 1 Diabetes mellitus and diabetic corneal neuropathy

Diabetes mellitus (DM), characterised by elevated levels of blood glucose resulting from defective insulin secretion and/or action, has emerged to become a major global public health problem (1). In 2021, 537 million adults were living with diabetes, and estimably 6.7 million adults have died because of DM or its complications (2). The estimated global cost of diabetes was projected to increase from US$1.31 trillion in 2015 to $2.1 trillion in 2030 (3). DM is associated with both macrovascular complications, such as cardiovascular disorders, and microvascular complications, including diabetic peripheral neuropathy (DPN) (4, 5). The manifestation of DPN in the cornea is referred to as diabetic corneal neuropathy, leading to diabetic keratopathy.

**Figure 1:**
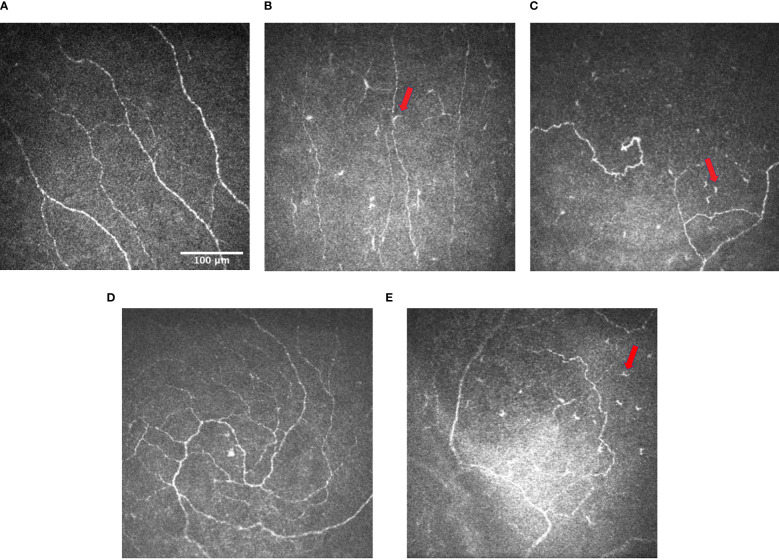
Representative IVCM images of **(A)** subbasal nerve plexus in normal controls (image taken from the subtemporal quadrant of the cornea of a middle-aged patient) (CNBD: 12.4992 no./mm^2^); **(B)** subbasal nerve plexus with decreased corneal nerve fiber length and density in patients with DM, and the presence of dendritic cells (arrows) (CNBD: 0 no./mm^2^) **(C)** subbasal nerve plexus with increased tortuosity and the presence of dendritic cells (arrows) in patients with DM (CNBD: 18.7488 no./mm^2^) **(D)** inferior whorl of corneal nerves in normal controls (CNBD: 43.7472 no./mm^2^); and **(E)** inferior whorl of corneal nerves in patients with DM showing the reduction in corneal nerve fiber length and density and the presence of dendritic cells (arrows) at the inferior whorl (CNBD: 6.2496 no./mm^2^). Images were produced *via* the Heidelberg retina tomograph (HRT) Corneal Module (Heidelberg Engineering, Heidelberg, Germany), laser scanning confocal microscopy.

DCN is characterized by changes in corneal nerve fibres and occurs in 47-64% of patients during their clinical course of DM (6, 7). When evaluating corneal nerve changes in DCN, *in-vivo* confocal microscopy (IVCM) has been considered the gold standard. *In vivo* cell imaging uses light reflected from within the tissue, gathering information to aid the recognition of inter- and intracellular details (8). Different from conventional microscopy where the image can be observed directly, confocal microscopes obtain increased resolution by limiting the illumination and observation systems to a single point. Hence, to reconstruct a full field of view and allow for “real-time” viewing, rapid scanning is used for IVCM (8, 9). IVCM produces high-resolution images at a cellular level with a magnification of 600-800 times, a lateral image resolution of 1-2μm, and an axial resolution of 5-10 μm (10). Post-imaging quantitative evaluations of corneal nerve plexus can be done manually, in a semi-automated manner, or a completely automated manner using certain analytic software (5, 11). Numerous studies have reported IVCM findings of reduced corneal nerve fibre density (CNFD), corneal nerve fibre length (CNFL), and corneal nerve branch density (CNBD) in patients with type 1 diabetes mellitus (T1D) or type 2 diabetes mellitus (T2D) (**Figures 1A, B**) (5). A reduction in nerve beading frequency is also observed, indicating a decrease in nerve metabolic activity and an increase in the risk of neuronal damage (7). In addition, patients with T1D or T2D present with an increase in nerve fibre tortuosity, reflecting a degenerative and subsequent attempted regenerative nerve response (**Figure 1C**) (12, 13). Besides nerve changes in the central and peripheral cornea, an earlier reduction in CNFL and CNBD of the subbasal inferior whorl of the corneal nerves, located in the inferonasal cornea, is also reported, serving as an imaging site for early detection of DCN (**Figures 1D, E**) (5, 7, 14). Moreover, patients with T1D have a lower corneal nerve fractal dimension (CNFrD) compared to control subjects, suggesting a less healthy and less evenly-distributed nerve fibre network in patients with T1D (7). Changes in the morphology and quantity of corneal dendritic cells (DCs) in diabetic corneas were also observed in several studies (15). However, the role and pathogenesis of the accumulation of the DCs have been not well understood.

## 2 Dendritic cells in normal corneas

DCs are bone marrow-derived antigen-presenting cells (APCs) that act as the initiator and modulator of immune responses (16). They are distinguished from other immune cell types through their cytoplasmic extensions (the dendrites), poor phagocytic activity, and the scarcity of their intracellular organelles (16). The most notable function of the DC family is to initiate primary T-lymphocyte-mediated immunity in response to an antigenic stimulus (17). This is achieved mainly through three functions of DCs: (a) capturing and presentation of antigens as sentinel cells; (b) migrating and binding to the antigen-specific T cells in lymphoid organs, and (c) activating T-cells and inducing their growth and proliferation (17).

### 2.1 Distributions of corneal dendritic cells

Naïve corneas were originally considered to lack the antigen-presenting system of DCs, contributing to their immune-privileged nature (18). However, more recent studies have shown a significant population of different subtypes of DCs residing in the cornea, with the number of which decreasing from the periphery towards the centre (19–22). Among the peripheral regions of the cornea, the inferior region has the highest density of DCs, followed by the superior region and the nasal region, while the temporal region has the lowest (22). In general, DCs can be subdivided into three main groups: the conventional DCs (cDCs), the plasmacytoid DCs (pDCs), and the monocyte-derived DCs (moDCs) (23). Such DCs subpopulations are defined based on their ontogeny, functional specialisation, and the requirement of specific transcription factors (TF) for the development (24). Different subtypes of corneal DCs are found in corneal epithelium and anterior stroma respectively (25, 26). Langerhans Cells (LCs), historically considered a subtype of conventional DCs (cDC), are observed in the periphery and centre of both human and murine corneal epithelium (25–29). However, the classification of LCs remains a topic with ongoing debate, since LCs were found to share properties with both DCs and macrophages. It has been argued by some that LCs may be considered a pecialized subset of tissue-resident macrophages based on their shared developmental origin (30, 31). Indeed, common DC precursors were found not to give rise to epidermal LCs. However, LCs share a remarkable number of functions with DCs, including migration to lymph nodes, and T-cell stimulation (31). The use of the term “LCs” has not been consistent across IVCM studies, and some other terms, such as APCs, dendritiform cells, or immune cells have also been used (28). Besides corneal epithelium, the anterior corneal stroma is also endowed with a different population of cDCs, namely the interstitial DCs. The interstitial DCs are primarily located in peripheral and paracentral regions of the anterior stroma with some toward the central anterior stroma in both murine and human cornea (25, 26, 29, 32). More recently, plasmacytoid dendritic cells (pDCs) have also been observed in the anterior stroma as well as epithelium in both the central and peripheral cornea of mice and human cadaver (29, 33–35).

### 2.2 Functions of corneal dendritic cells

Normally, mature DCs have developed dendrites that are absent in immature DCs (28). Unlike mature DCs, immature DCs lack the requisite accessory signals for T-cell activation, such as CD40, CD80, and CD86. To induce maturation of the dormant immature DCs, signals in the extracellular milieu through inflammatory mediators are needed (32). The distribution of corneal DCs at different maturation stages in the human cornea remains an issue of ongoing discussion. Some are consistent with the murine studies, which reported immature LCs in the centre of corneal epithelium, and both mature and immature LCs in the peripheral corneal epithelium (22, 27). Others demonstrated few mature LCs and interstitial DCs in epithelium and stroma respectively in both the peripheral and central cornea (25). The differences may have arisen from several reasons, potentially including different maturation markers and different models (*in-vivo* or ex-vivo) used (25, 27).

DCs serve both immunological and non-immunological roles in human cornea. The primary function of DCs in the cornea is to induce and amplify immunoinflammatory responses (18, 36). During the inflammatory process triggered by infection or allergy, the release of pro-inflammatory cytokines, such as interleukin (IL)-1, tumour necrosis factor (TNF)-α, CD40L, and lipopolysaccharide, or heat-shock proteins from dying cells, facilitates the activation of LCs/DCs in the cornea (20, 21). Resultingly, surface expression of co-stimulatory molecules (CD80/CD86) and CD40 is increased by DCs/LCs in the peripheral cornea, as well as acquired *de novo* by immature DCs/LCs in the central cornea (37). The activated corneal LCs/DCs function as APCs by transporting the antigens to lymphoid organs and presenting them to effector or memory T cells, priming the T cells for the antigen-specific adaptive immune response (18, 36, 37). Resident corneal DCs are considered long-lived, though it is still uncertain whether during the steady state, the corneal DCs self-regenerate through mitosis, emerge from tissue-resident precursors, or are recruited from the circulating blood (38–40). Nonetheless, in the presence of inflammatory stimuli and increased chemokine/cytokine levels in the cornea, corneal DCs are increased, at least partially through the recruitment of DC precursors from the blood (26, 38).

The non-immunological function of LCs/DCs is associated with tissue repair, through partnering with surrounding corneal epithelial cells. Upon injury, corneal intraepithelial LCs/DCs are activated either directly through recognition of danger signals, or indirectly from cytokines and chemokines secreted by epithelial cells in the injury site. The activated LCs/DCs modulate the migration, proliferation, and survival of epithelial cells in the wounding area *via* either cell-to-cell contact or the release of survival and growth factors (41). The epithelial cells, in turn, further activate corneal LCs/DCs and recruit them into the wound bed *via* epithelia-generated mediators (41).

It Is worth noting that although DCs and macrophages were historically regarded as two distinct types of immune cells, the classifications of DCs and macrophages have recently been challenged and remain a topic of ongoing discussion (42, 43). Due to some shared surface markers and functional parameters between renal DCs and macrophages in both acute renal injury and chronic immune-mediated kidney disease (42–46). It was argued that the functional and phenotypic definitions of these two cell types, especially in the kidney, overlap greatly (42). Therefore, an improved classification system may be needed to better facilitate future research work (42, 44).

## 3 *In-vivo* confocal microscopy (IVCM) evaluation on corneal DCs

**Figure 2:**
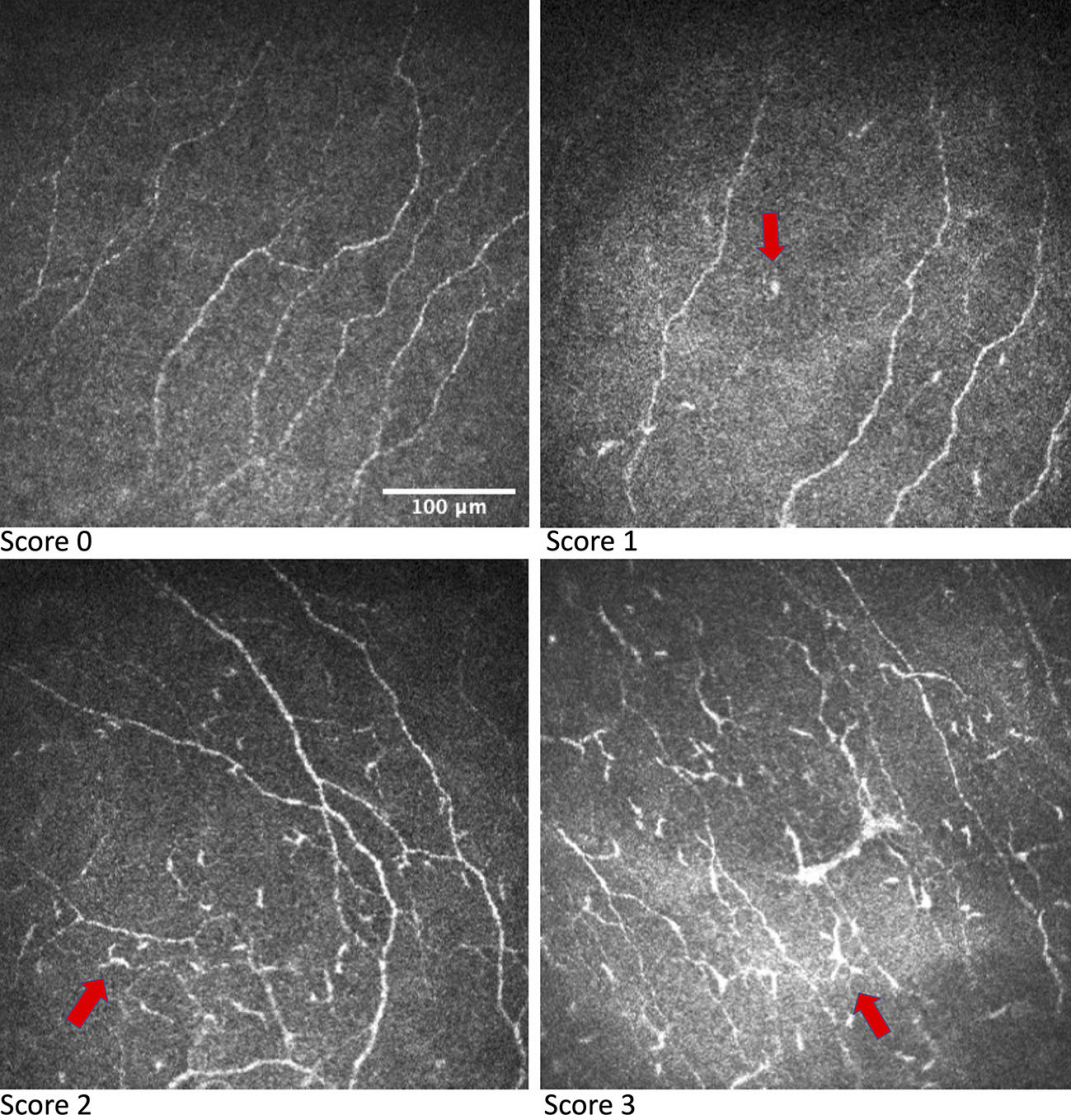
IVCM images showing grading of DC morphology. DCs are indicated by the arrow in respective IVCM images. Images were produced *via* the Heidelberg retina tomograph (HRT) Corneal Module (Heidelberg Engineering, Heidelberg, Germany), laser scanning confocal microscopy.

As IVCM can provide images at the cellular level, it has been used to evaluate the DC morphology and distribution (27, 47). Using IVCM, changes in corneal DCs have been observed in ocular surface diseases including dry eye disease and infectious keratitis, as well as systemic disorders including DM, multiple sclerosis, rheumatoid arthritis, ankylosing spondylitis, and systemic lupus erythematosus (15, 48–51). On IVCM evaluation, corneal epithelial DCs present as bright corpuscular particles and a diameter of up to 15μm (27). The presence of Birbeck granules, a type of cytoplasmic marker granules, distinguishes LCs from other DCs (27). Currently, phenotypic classification of corneal epithelial DCs is achieved mainly through morphological differences (49). The DCs morphology can be evaluated according to a 0-3 scale based on the size of the dendrites compared to the largest diameter of the cell body (**Figure 2**): A score 0 indicates an absence of DCs; a score 1 indicates the presence of DCs without processes; a score 2 indicates the presence of DCs with small processes, the length of which does not exceed the largest diameter of the cell body; a score 3 indicates the presence of DCs with long processes, the length of which exceeds the largest diameter of the cell body (48, 52). Longer processes and smaller cell bodies in DCs indicate a higher level of maturation and potential activity (48, 52).

## 4 Search strategy and selection criteria

The authors conducted a search on the online database PubMed Central, Google Scholar, and Science Direct for relevant articles that describe the changes in corneal dendritic cells in subjects with T1D/T2D or the relationship between corneal DCs and clinical or corneal imaging parameters in patients with T1D/T2D.

Articles were included up to May 2022. Keywords included but were not limited to “diabetes” AND “hyperglycaemia” AND “corneal dendritic cells” OR “corneal Langerhans cells” AND “corneal nerve” AND “corneal neuropathy” AND “corneal epithelial cells” AND “ag”“ AND “diabetes duration” AND “blood metabolic profile”. Our review only examined papers written in English, and we restricted the date of publication to the most recent ten years as much as possible. We have also extracted relevant articles from the bibliographies of the existing articles. The authors then manually screened the abstracts and shortlisted papers based on our inclusion criteria. The full-text version of all selected articles was further examined.

## 5 Changes in corneal dendritic cells in patients with DM

DM affects multiple ocular tissues, including the cornea (49). Studies have examined the changes in DCs, including the DC density, maturation stages, as well as relationships between the corneal DCs, corneal nerves, and corneal epithelium, in subjects with T1D/T2D (15, 53–60). Corneal DCs may serve as a biomarker for DM-associated ocular surface complications, such as diabetic corneal neuropathy (54).

### 5.1 Changes of corneal DC density in DM

Changes in DCs density in subjects with T1D/T2D have been investigated and remain a topic of ongoing discussion. The majority of animal and clinical studies reported an increase in DCs density in subjects with T1D/T2D, while a few presented the opposite (15, 53–59). The literature review on this topic is summarised in **Table 1**.

**Table 1:** Studies reporting the changes in DC density in DM.

Authors	Study population	DC quantification technique	Findings
Literature reporting increased DC density in DM:			
Leppin et al. (15)	Streptozotocin (STZ)-induced T1D mice and Lep^ob/ob^ mice with T2D	IVCM and *in-vitro* corneal whole-mounts confocal microscopy (WMCM)	Both STZ-induced mice and Lep**^ob/ob^** mice experienced increased corneal DC density.
Colorado et al. (53)	Patients with T1D	Time-lapsed IVCM	A higher density of DC without dendrites was observed in subjects with T1D compared to healthy controls.
D**’**Onofrio et al. (54)	Patients with T1D, T2D, or latent autoimmune diabetes of adults (LADA)	Laser scanning IVCM	A higher DC density was observed in patients with T1D, T2D, and LADA compared to controls.
Tavakoli et al. (55)	Patients with T1D/T2D and varying severities of diabetic peripheral neuropathy	IVCM	A significant increase in DC density was observed in patients with T1D/T2D and no or mild peripheral neuropathy A decrease in DC density was reported in patients with T1D/T2D and moderate or severe peripheral neuropathy, yet DC density still remained higher than control values.
Ferdousi et al. (56)	Children with T1D	IVCM	A significantly higher total DC density was observed in individuals with T1D compared to controls.
Qu et al. (58)	Patients with T2D diagnosed with corneal punctate epitheliopathy	IVCM	A significantly higher LC density was reported in punctate epitheliopathy patients with T2D compared to punctate epitheliopathy resulting from other causes.
Qu et al. (57)	Patients with T2D without and with cornea fluorescein staining	IVCM	A significantly higher LC density existed in T2D patients compared to healthy controls in all corneal areas. A significantly higher LC density was reported in T2D patients with punctate epitheliopathy compared to those without in the central and inferior zones of the cornea.
Literature reporting decreased DC density in DM:			
Gao et al. (59)	STZ-induced T1D mice	Whole-mount confocal microscopy (WMCM)	A reduced number of intraepithelial DCs was reported in diabetic corneas compared to non-diabetic corneas.
Literature reporting no significant change in DC density in DM:			
Chao et al. (61)	Patients with prediabetes or T2D	IVCM	No significant difference in DC density among patients with prediabetes, T2D, and healthy controls was observed.

When mice models are used to investigate T1D, streptozotocin (STZ) is commonly used to induce hyperglycemia through the destruction of pancreatic β-cells (15, 59, 62). *Via* corneal IVCM and corneal *in vitro* whole-mounts confocal microscopy (WMCM), a constant increase of corneal DC density over 9 weeks upon diabetes induction was observed in one murine study (15). Contrastingly, another study using WMCM observed a significantly lower number of DCs in corneas from STZ-induced mice with T1D compared to controls (59). On the other hand, Lep^ob/ob^ mice are often used as mouse models of T2D (62). One murine study investigating mice with T2D reported that the dendritic cell density in type 2 diabetic mice was 3-fold higher than in non-diabetic mice (15).

In clinical studies, the DC density was quantified in patients with T1D or T2D using IVCM. Compared to healthy controls, significantly higher DC density was observed in patients with T1D or T2D, as well as in patients with T1D/T2D and peripheral/somatic neuropathy or corneal punctate epitheliopathy (53–58). Among patients with T1D/T2D and peripheral/somatic neuropathy, DC density was significantly higher in patients with no or mild peripheral/somatic neuropathy compared to non-diabetic controls. However, with the progression of peripheral neuropathy, the DC density was reduced in patients with T1D/T2D and moderate or severe neuropathy, though remained above control values. The authors proposed that DCs might be only involved in the early phases of nerve degeneration whereas the later phase of nerve damage in DM may be maintained by other factors, including glucose neurotoxicity (55, 63). The DC density in patients with T2D and corneal punctate epitheliopathy was also investigated (57, 58). A significantly higher DC density in all corneal areas was observed in both groups of type 2 diabetic patients with and without punctate epitheliopathy compared to healthy controls. In another study where the authors compared the DC density in patients with punctate epitheliopathy resulting from T2D or other causes, a significantly higher DC density was found in the former group, suggesting an association between T2D status and DC density (58).

There are several mechanisms proposed for the increase of DC density in DM populations. Though DM is characterised by elevated levels of blood glucose, it is suggested that the increase in DC might be unrelated to hyperglycaemia as no correlation between DC density and glycaemic control was observed (57). Instead, the increase of DCs in patients with T1D/T2D may be deemed as a cellular response to inflammation. Diabetes, especially T2D, has been suggested to be a pro-inflammatory cytokine-associated disease, involving both the innate and adaptive immune systems (60). There are several pathogeneses involved in the inflammatory state of T2D, including tissue hypoxia, cell death of expanding adipose tissue, activation of interleukins, and nuclear factor (NF)-κB pathways, contributing to the recruitment and activation of immune cells (5, 7, 64). For example, the NF-κB signaling pathway may be activated *via* the interaction of advanced glycation end-products (AGEs) and its cognate receptor for advanced glycation end-products (RAGE), subsequently promoting the secretion of TNF-α, IL-1β, IL-6, and other pro-inflammatory cytokines (65–68). Significantly increased levels of various AGEs compounds have been reported in both type 1 and type 2 diabetic patients, resulting from non-enzymatic glycation and oxidation of proteins and lipids (65, 69–75). The mechanism for the increased DC density is supported by the observations that corneal DC infiltration and maturation are induced when inflammatory stimuli like electric cautery, lipopolysaccharide, and tumour necrosis factor-α are applied to the ocular surface (26, 76). Findings by another study are also in line with this proposed mechanism where the DC density in the cornea increased by a factor of approximately eight during immune-mediated corneal inflammation secondary to an infection, allergy, or corneal graft rejection (77).

On the contrary, some literature showed the opposite findings in which decreased corneal DCs were observed in both animal models and patients with T1D/T2D (59, 78, 79). One proposed explanation is that prolonged exposure to hyperglycaemia may cause DC apoptosis, reducing the DCs density (80). Similar observations in other immune cells, such as increased apoptosis in neutrophils as well as impaired antigen presentation by monocytes, have also been reported under chronic hyperglycaemic conditions (81). Such observation might also be attributed to the different imaging techniques used.

### 5.2 Changes in maturation stages of corneal DCs in DM

Besides the density changes, changes in the maturation stages of DCs are reported in DM. Through wide-area three-dimensional mosaic projections of the corneal subbasal nerve plexus, a doubling in mature DCs (mDCs) proportion, as well as a proportional decrease in immature DCs (imDCs), were observed in patients with T2D (60). This finding suggests that the maturation of corneal DCs occurs as T2D develops. This is also supported by another study where the authors reported a higher percentage of patients with T1D/T2D/latent autoimmune diabetes of adults (LADA) (95%) with mature DCs in their central cornea compared to healthy controls (65%), while immature DCs can be found in all participants, including patients with T1D/T2D/LADA and controls (54).

It is proposed that tumour necrosis factor receptor super family member 9 (TNFRSF9) acts as a key contributor to the changes in the maturation stages of corneal DCs, by promoting the maturation and survival of DCs (60). Out of 92 plasma proteins analysed in a clinical study, TNFRSF9 was associated with the observed maturation of DCs from an immature to mature antigen-presenting phenotype. There was a significant association between TNFRSF9 and the proportion of mDC, and TNFRSF9 was also inversely correlated with the imDC proportion (60). TNFRSF9 is found to be expressed on immune cells including activated and regulatory T-cells and activated natural killer (NK) cells (82–84). Hence, when T cells are activated with the onset of T2D, it subsequently induces the expression of TNFRSF9, which further promotes the maturation of the DCs (60). Besides TNFRSF9, the involvement of AGEs in regulating the maturation of DCs has also been reported in both *in vitro* studies of human tissue and *in vivo* studies of diabetic mice with myocardial infarction (85, 86). The maturation of DCs in patients with T1D/T2D may be induced by the increased level of AGE through promoting the expressions of scavenger receptor-A (SR-A) and RAGE, *via* the Jnk pathway. Such a mechanism has been proposed in patients with atherosclerosis (86).

## 6 Relationship between corneal DCs and clinical or corneal imaging parameters

Studies have reported the associations between the changes in corneal DCs and various clinical or corneal nerve imaging parameters, including age, corneal nerve status, and blood metabolic parameters (15, 53–58, 87). Such associations may contribute to the current understanding of DM, further helping the development of diagnostic measures and biomarkers, as well as preventive and therapeutic strategies (54, 88, 89).

### 6.1 Relationship between corneal DCs and age

In healthy individuals, corneal DC density was reported to be independent of age by a meta-regression analysis (90). On the contrary, a significant and positive correlation between the DC density and age was observed in patients with T1D/T2D (55) (56). Moreover, specific to children with T1D, a significant positive correlation was found between the pubertal stage and the mature DC density, immature DC density, as well as to total DC density (56). These findings indicate that age may be a potential differential risk of DM and DM-associated ocular surface complications (56).

Besides the DC density, the age of the patients with T1D was also reported to be inversely correlated with the displacement of DCs without dendrites (woDCs), as well as the woDCs’ persistence ratio (53). DC displacement was calculated as the straight-line distance between the start and end positions of a DC divided by the total time of movement. The authors further proposed that faster DC movements represent healthier DC behaviour and that reduced DC migration in older patients may contribute to age-associated immune dysfunction (53, 91, 92).

However, it is not entirely known how or whether the observed correlations between age and the corneal DC parameters in patients with T1D/T2D are involved or if they are influenced by the pathogenesis of diabetes. Aging has been linked to diabetes through several mechanisms, including age-associated insulin resistance and age-dependent disruption of insulin production (93, 94). Given that both the prevalence and incidence of T2D have been reported to increase dramatically as a function of age, further understanding of the mechanisms underpinning this differential risk is of great importance in the development of age-appropriate preventive and therapeutic strategies (88, 89).

### 6.2 Associations between corneal DCs and corneal nerves

Diabetes may perturb the interaction between DCs and other structures, especially corneal nerves (80). In the confocal images of mouse corneas stained with CD11c (inflammatory marker) and β-tubulin 3 (neuronal marker), intimate contacts between the DC body and its processes with sensory nerve endings were observed (59). It has also been demonstrated that DCs may be involved in diabetic nerve degeneration, yet whether DCs are neuroprotective or neurotoxic remains unclear with contrasting findings (49, 55).

Corneal nerve degeneration in DM may be associated with an increased DCs density (15). In STZ-induced type 1 diabetic mice, a significant negative correlation was reported between the corneal nerve fiber length and DC density (15). It was also observed that the density of DCs was higher in patients with T1D/T2D and no or mild peripheral neuropathy compared to those with moderate and severe peripheral neuropathy. The authors then proposed that DCs might be involved in the initial phase of nerve damage (55). This theory was further evidenced by the findings of other clinical studies. In patients with T1D/T2D, a significant negative correlation between increased DC density and corneal nerve fibre density, branch density, as well as fibre length was observed, suggesting a potential interaction between activated DCs and corneal nerve fibre degeneration (54, 58). Moreover, in adults with T1D or T2D with or without punctate epitheliopathy, a significant negative correlation was reported between the corneal nerve fiber length and DC density, specifically immature DC density for type 1 diabetic patients (15, 54, 57–59). An inverse correlation between the total DC density and corneal nerve total branch density was also reported in patients with T1D (54). In the immune-neuron crosstalk between nerves and DCs, cells from the neuroendocrine systems recognise the cytokines produced by immune cells. Reciprocally, the immune cells recognise the neurotransmitters and neuropeptides produced by the corneal nerves (95). It is speculated that the DC-nerve interaction in the cornea may be analogous to the neuro-immune axis in the skin and the gut (49, 80). For example, it was demonstrated that calcitonin gene-related peptide (CGRP)-containing nerve fibres were intimately associated with DCs and that CGRP could inhibit antigen presentation by epidermal DCs (96). The tolerogenic and immunomodulatory effects of many neuropeptides have also been previously indicated, which, in their absence due to damages to corneal nerves could lead to enhanced immune response, including increased DC density (97).

Contrary to the previous discussion, some studies found that nerve degeneration in DM may be associated with reduced DCs density (59). In STZ-induced type 1 diabetic mice with corneal epithelial debridement wounds, a reduced number of infiltrating DCs, as well as delayed sensory nerve regeneration, were observed (59). Though these observations were opposite from the findings of most other studies reporting on the same matter, the authors suggested a possible explanation (15, 54, 58). It is postulated that DCs may mediate corneal nerve innervation and regeneration through ciliary neurotrophic factor (CNTF). In the cornea, DCs are the major source of CNTF (59). It was demonstrated in mice with T1D that injection of CNTF-neutralising antibodies delayed nerve-ending regeneration, while exogenous CNTF accelerated nerve regeneration in corneas with local DCs depleted (59). Moreover, blocking the CNTF-specific receptor, CNTFα, induced corneal sensory nerve degeneration and delayed nerve regeneration, demonstrating the importance of CNTFα in the maintenance and regeneration of subbasal nerve plexus (59). Hence, in the case of the STZ-induced type 1 diabetic mice with corneal epithelial debridement wounds, decreased number of DCs on the cornea would lead to a decreased CNTF level, impairing corneal sensory nerve innervation and regeneration (59). Besides the involvement of CNTF, DCs may also be involved in the regeneration of neurons through the clearance of axonal debris. It was suggested that the clearance of axonal debris is a critical process in axonal regeneration in the peripheral nervous system (98). Besides murine studies, a clinical study has also reported similar observations where in children with T1D, a significant positive correlation was observed between the density of mature DCs and the corneal nerve fiber density (56).

However, the relationship between the DC density and corneal nerve parameters in subjects with T1D/T2D remains a topic for more investigation. There were also studies reporting no significant correlation to exist between the DC density and corneal nerve morphology in either T1D or T2D (54, 55). The analysis of the relationship may be confounded by several factors, such as variations in the type, stage, and duration of DM. It is also possible that corneal nerve fibre changes and DC density are different and independent phenomena that occur coincidentally at the same time, and other cells also play a role (15). For example, vascularisation that develops after denervation may also lead to the influx of DCs (15). Moreover, in patients with T1D/T2D, increased levels of AGE/RAGE signaling in neurons may induce the activation of inflammatory and oxidative stress pathways, including the NF-κB pathway, potentially causing damage and death of neuronal cells (99–101).

The associations reported may further help explore surrogate imaging markers for diabetic corneal neuropathy (54). For example, a significant correlation between DC density and the severity of diabetic peripheral neuropathy has been described (55). Moreover, the reported associations between the DC density and various nerve parameters may provide evidence for a potential therapeutic strategy to promote corneal nerve regeneration. For example, given the fact that DCs are the major source of CNTF, using DCs as therapeutic targets for the repair of injured corneal nerves in patients with T1D/T2D may open a new avenue for treatment (49, 59).

The literature review on the association between corneal DCs and corneal nerve parameters is summarised in **Table 2**.

**Table 2:** Studies reporting on the correlation between DC density and various corneal nerve imaging parameters.

Authors	Study population	Nerve imaging parameters assessed	Findings
Ferdousi et al. (56)	Children with T1D (Age: 14.6 ± 2.5; Diabetes duration: 9.1 ± 2.7 years)	Corneal nerve fibre density	↑ density of mature DCs density, ↑ corneal nerve fibre density (r = 0.2, P = 0.01) in patients with T1D
D**’**Onofrio et al. (54)	Patients with T1D (Age: 53.3 ± 11.7; Diabetes duration: 19.4 ± 7.6 years), T2D (Age: 57.7 ± 7.5; Diabetes duration: 15.1 ± 4.9 years), or latent autoimmune diabetes of adults (LADA) (Age: 50.5 ± 11.5; Diabetes duration: 11.6 ± 9.6 years)	Corneal nerve fibre density	No significant correlation between DC density and corneal nerve fibre density in patients with T1D, T2D, or LADA.
D**’**Onofrio et al. (54)	Patients with T1D (Age: 53.3 ± 11.7; Diabetes duration: 19.4 ± 7.6 years), T2D (Age: 57.7 ± 7.5; Diabetes duration: 15.1 ± 4.9 years), or latent autoimmune diabetes of adults (LADA) (Age: 50.5 ± 11.5; Diabetes duration: 11.6 ± 9.6 years)	Corneal nerve branch density	↑ mature DC density, ↓ corneal nerve branch density (r = –0.5; P = 0.008); ↑ immature DC density, ↓ corneal nerve branch density (r = –0.4; P = 0.02); ↑ total DC density, ↓ corneal nerve branch density (r = –0.5; P = 0.01) DC density in patients with T1D but not in patients with T2D and LADA.
Ferdousi et al. (56)	Children with T1D (Age: 14.6 ± 2.5; Diabetes duration: 9.1 ± 2.7 years)	Corneal nerve branch density	No significant correlation between DC density and corneal nerve branch density in children with T1D.
D**’**Onofrio et al. (54)	Patients with T1D (Age: 53.3 ± 11.7; Diabetes duration: 19.4 ± 7.6 years), T2D (Age: 57.7 ± 7.5; Diabetes duration: 15.1 ± 4.9 years), or latent autoimmune diabetes of adults (LADA) (Age: 50.5 ± 11.5; Diabetes duration: 11.6 ± 9.6 years)	Corneal nerve fibre length	↑ immature DC density, ↓ corneal nerve fibre length (r = –0.4; P = 0.03) in patients with T1D but not in patients with T2D and LADA.
Qu et al. (58)	Patients with T2D diagnosed with corneal punctate epitheliopathy (Age: 59.8 ± 11.6; Diabetes duration: 13.4 ± 8.30 years)	Corneal nerve fibre length	↑ DC density, ↓ corneal nerve fibre length (r = 0.350; R2 = 0.1225; P = 0.034) in patients with T2D diagnosed with corneal punctate epitheliopathy
Qu et al. (57)	Patients with T2D without (Age: 60.51 ± 8.37; Diabetes duration: 13.40 ± 8.30 years) and with (Age: 63.75 ± 10.91; Diabetes duration: 13.90 ± 5.20 years) cornea fluorescein staining	Corneal nerve fibre length	↑DC density, ↓ corneal nerve fibre length in all corneal zones except the superior zone in patients with T2D.
Leppin et al. (15)	Streptozotocin (STZ)-induced T1D mice	Corneal nerve fibre length	↑ DC density, ↓ corneal nerve fibre length existed in STZ-induced diabetic mice. No such correlation was observed in non-diabetic controls.
Ferdousi et al. (56)	Children with T1D (Age: 14.6 ± 2.5; Diabetes duration: 9.1 ± 2.7 years)	Corneal nerve fibre length	No significant correlation between DC density and corneal nerve fibre length in children with T1D

### 6.3 Interaction between corneal DCs and corneal epithelium in DM

Besides the interaction with the nerve, the interaction between DCs and epithelial cells may be perturbed in subjects with T1D/T2D, potentially affecting the corneal wound healing (80).

In both murine and clinical studies, it was observed that subjects with T1D/T2D had delayed corneal wound healing compared to healthy controls (59, 102–105). A decrease in basal epithelial cell (BEC) density in patients with T2D has also been reported by several clinical studies (57, 106, 107). Furthermore, a negative correlation between the BEC density and DC density in the cornea was observed in patients with T2D (57). Hence, it was speculated that DCs may be involved in the early stages of BEC proliferation and differentiation in DM (57). Epithelial wound closure requires cell reverse differentiation of wing cells to basal cell like cells, cell migration, and cell proliferation to replenish the lost cells (108, 109). Besides epithelial cells, immune cells, such as DCs, were also directly involved in accelerating epithelial wound healing (80). The anatomical proximity and structural intertwinements between DCs and epithelial cells have led to the suggestion that corneal epithelial cells and corneal intra-epithelial DCs interact with each other to form coordinated actions against adverse challenges, such as tissue injury and infection (41, 80). It was demonstrated in non-diabetic corneas that migratory epithelial cells during wound healing would express an elevated level of DC-targeting cytokines, to activate DCs around the injury site (41). Reciprocally, DCs would secrete growth factors, cytokines, and/or through cell-to-cell contact to facilitate migration and proliferation of epithelial cells, modulating wound healing (41, 59). However, the specific role of DCs in delayed epithelial wound healing in patients T1D/T2D remains unclear, and several explanations have been proposed (41).

One explanation is that prolonged corneal wound healing response in subjects with T1D/T2D may lead to increased recruitment of DCs to the corneal wound through chemokines released by the injured site (41, 103). Several factors were reported to contribute to the delayed recovery of corneal epithelial wounds in DM, including nerve degeneration, accumulation of AGEs, and direct damage caused by hyperglycaemia to the corneal epithelial basement membrane (110). In particular, it was suggested that AGEs may delay corneal epithelial wound healing through the production of reactive oxygen species (111). In a wounded cornea, the corneal epithelial cells can further facilitate wound healing through the release of various cytokines, including the C-X-C motif chemokine ligand 10 (CXCL10) (41). CXCL10 acts as a chemokine to DCs, activated T cells, and NK cells, and it was reported to be highly expressed in migrating epithelial during corneal wound healing (41, 112). Hence, it was suggested that epithelia-released CXCL10 may facilitate the recruitment of resident corneal epithelial DCs and even the circulating DCs to the wound bed in the cornea (41). It is possible that, in diabetic cornea where the wound healing process is altered and prolonged, more chemokines may be released by the epithelia, resulting in increased recruitment of DCs into the cornea (103, 113). Similarly, clinical studies on the epidermis of diabetic foot ulcers have reported an accumulation of DCs at the edge of diabetic foot ulcers (113, 114).

Contrary to the aforementioned explanation of increased DCs in diabetic wound healing, a murine study examining subjects with T1D has reported a decreased number of infiltrating DCs in diabetic healing cornea compared to healthy controls. It was proposed that such a decrease in DCs population may hinder the proliferation of the epithelial cells, contributing to the impaired wound healing process (59, 115). As discussed previously, CNTF originates from DCs and is involved in sensory nerve survival and regeneration (59). Recently, CNTF was also discovered to be able to promote epithelial wound healing by stimulating the mitogenic activation of corneal epithelial stem/progenitor cells (115). In the corneas of mice with T1D, the level of CNTF was significantly downregulated, potentially due to the decreased infiltrating DCs population, contributing to the impaired proliferation of epithelial cells during wound healing in diabetic corneas (59, 115).

### 6.4 Correlations between corneal DCs and blood metabolomic profiles

Associations between the DC density and several metabolic parameters, including lipid profiles, glycaemic control, as well as renal function, have also been assessed in patients with T1D/T2D, as shown in **Table 3** (55, 56, 87).

**Table 3:** Studies reporting on the correlation between corneal DC parameters and blood metabolic parameters.

Author (year)	Study population	Blood metabolic parameters assessed	Findings
Colorado et al. (87)	Patients with T1D (Age: 55.0 ± 11.0; Diabetes duration: 29 ± 14 years)	Lipid profile	↑ corneal DCs without dendrites (woDCs) density, ↑ the HDL level (r = 0.59, p = 0.007); ↑ corneal DCs without dendrites (woDCs) density, ↓ the triglyceride level (r = −0.61, p = 0.005); ↑ rounded corneal DC density, ↓ the HDL level (r = −0.54, p = 0.007) in patients with T1D.
Colorado et al. (87)	Patients with T1D (Age: 55.0 ± 11.0; Diabetes duration: 29 ± 14 years)	Glycaemic control	No significant association between HbA1c and corneal DC density as well as DC dynamics in patients with T1D.
Tavakoli et al. (55)	Patients with T1D/T2D and varying severities of peripheral neuropathy (Age: 58 ± 1; Diabetes duration: 15 ± 1 years)	Glycaemic control	No significant correlation between DC density and HbA1c.
Ferdousi et al. (56)	Children with T1D (Age: 14.6 ± 2.5; Diabetes duration: 9.1 ± 2.7 years)	Glycaemic control	No significant correlation between DC density and HbA1c
Colorado et al. (87)	Patients with T1D (Age: 55.0 ± 11.0; Diabetes duration: 29 ± 14 years)	Renal function	↑ displacement of corneal DCs, ↑ eGFR (r = 0.74, p < 0.001); ↑ trajectory of corneal DCs, ↑ eGFR (r = 0.48, p = 0.031); ↑ persistency of corneal DCs, ↑ eGFR (r = 0.58, p = 0.008) in patients with T1D.

In patients with T1D, significant associations were found between the DC density and lipid parameters (87). The density of corneal DCs without dendrites (woDCs) was positively correlated with the HDL cholesterol level and was inversely correlated with the triglycerides level (87). Such observations suggest that woDC may be associated with better health since both a higher HDL level and a lower triglyceride level potentially indicate lower cardiovascular risks (116, 117). However, another clinical study demonstrated that rounded corneal DC density was correlated inversely with the HDL level in patients with T1D (87). Such disparity reported may signal that different DC subsets exert different immune activities on the cornea in patients with T1D (87).

For the renal function of patients with T1D, a significant positive correlation was detected between the eGFR and the displacement, trajectory, and persistency of corneal DCs in patients with T1D (87). These three parameters are indicative of DCs’ mobilisation capacities which are critical for the role of the DCs in activating and mediating immune responses (87, 118). It has been proposed that the resident DCs in the cornea may function similarly to those in the kidney. eGFR was also negatively correlated with the number of DCs in the kidney for both healthy individuals and those with chronic kidney disease (119).

Contrasting to lipid parameters and renal function, glycaemic control has not been reported to be significantly associated with DCs parameters (density and dynamics) (55, 56, 87). The increase in DC density observed in patients with T1D/T2D may be independent of hyperglycaemia (55).

## 7 Future work

The changes in corneal DCs in patients with T1D/T2D have attracted much attention and discussion in recent years. Despite the current progress toward understanding the DC changes and underlying mechanisms, many questions remain and are to be addressed.

Firstly, continued improvement in imaging technologies, as well as the identification and quantification techniques used for corneal DCs on IVCM images are required, to ensure accurate analysis across the studies. Secondly, it is possible that the identification and characterisation of corneal DCs *in vivo* may be further refined, to contribute to a deeper understanding of corneal DCs changes, as well as the roles played by corneal DCs in the diabetic corneas (49). Thirdly, DCs, corneal nerves, and corneal epithelium were previously considered to form an “epineuroimmune” function unit (80). However, it remains unclear which of the three components is the initial “sentinel” that detects the physiological changes in patients with T1D/T2D and subsequently induces the changes in the other two components of the “epineuroimmune” function unit. Furthermore, the initial “trigger” (e.g. hyperglycaemia, intracellular reactive oxygen species, or extracellular AGEs) in the diabetic cornea that causes the physiological and functional changes in the “epineuroimmune” function unit also requires elucidation (80). Hence, further mechanistic studies are needed to define the basis of the changes in the “epineuroimmune” function unit in the diabetic cornea, potentially adding value to the development of preventive and treatment strategies for DM-associated ocular surface complications (55).

## 8 Conclusions

This article has reviewed current clinical and animal studies reporting the changes in corneal DCs in diabetic corneas, as well as the potential mechanisms underlying the changes. For the changes in DC density, the majority of animal and clinical studies reported an increase in corneal DCs density in DM, while a few presented the opposite (15, 53–59). The increase in DC density may be explained as a cellular response to inflammation while the decreased density may be explained as a result of apoptosis caused by prolonged exposure to hyperglycaemia (60, 80). The maturation of corneal DCs in tandem with the disease course of T2D was indicated (60). DCs were also found to be involved in diabetic nerve degeneration, yet whether DCs are neuroprotective or neurotoxic remains unclear with contrasting findings (49, 55). The association between increased DCs density and corneal nerve degeneration in DM may be explained by an enhanced immune response caused by the absence of tolerogenic and immunomodulatory neuropeptides following corneal nerve damage (95). On the other hand, the association between decreased DCs density and corneal nerve degeneration in diabetic corneas may be explained by the decreased CNTF level expressed by the corneal DCs (59). Corneal DCs are also involved in delayed epithelial wound healing in diabetic corneas (80). One suggested mechanism is that prolonged corneal wound healing response leads to increased recruitment of DCs to the corneal wound bed through chemokines released by epithelia around the injury site (41, 103). We also further reviewed the association between the changes in the corneal DCs and various clinical or corneal nerve imaging parameters, including age, corneal nerve status, and metabolic parameters (15, 53–58, 87). Such associations contribute to our current understanding of DM-associated ocular surface complications, potentially further assisting the development of diagnostic, preventive, and therapeutic strategies.

## Author contributions

FL and LYC were responsible for conducting the search, screening potentially eligible studies, and writing the manuscript. CL was responsible for the synthesis of images. IL and ML were involved in conducting the search. LYC provided the direction of the review and overall supervision of this manuscript.
